# Identification of Conserved and Novel MicroRNAs during Tail Regeneration in the Mexican Axolotl

**DOI:** 10.3390/ijms160922046

**Published:** 2015-09-11

**Authors:** Micah D. Gearhart, Jami R. Erickson, Andrew Walsh, Karen Echeverri

**Affiliations:** 1 Department of Genetics, Cell Biology and Development, University of Minnesota, Minneapolis, MN 55455, USA; E-Mails: gearh006@umn.edu (M.D.G.); eric2843@umn.edu (J.R.E.); 2 Cenix BioScience GmbH, Dresden 01307, Germany; E-Mail: paeruginosa@hotmail.com; 3 Sitools Biotech GmbH, Planegg-Martinsried 82152, Germany

**Keywords:** axolotl, regeneration, microRNAs

## Abstract

The Mexican axolotl salamander (*Ambystoma mexicanum*) is one member of a select group of vertebrate animals that have retained the amazing ability to regenerate multiple body parts. In addition to being an important model system for regeneration, the axolotl has also contributed extensively to studies of basic development. While many genes known to play key roles during development have now been implicated in various forms of regeneration, much of the regulatory apparatus controlling the underlying molecular circuitry remains unknown. In recent years, microRNAs have been identified as key regulators of gene expression during development, in many diseases and also, increasingly, in regeneration. Here, we have used deep sequencing combined with qRT-PCR to undertake a comprehensive identification of microRNAs involved in regulating regeneration in the axolotl. Specifically, among the microRNAs that we have found to be expressed in axolotl tissues, we have identified 4564 microRNA families known to be widely conserved among vertebrates, as well as 59,811 reads of putative novel microRNAs. These findings support the hypothesis that microRNAs play key roles in managing the precise spatial and temporal patterns of gene expression that ensures the correct regeneration of missing tissues.

## 1. Introduction

Regeneration is defined as the regrowth or restoration of lost tissue. Mammals, unfortunately, have very limited regenerative wound healing capacities, addressing only small portions of liver, peripheral nerve damage and muscle through abilities that decline markedly with age. Salamanders, however, stand as the champions in this field, able to regenerate with full structural accuracy and functionality a long list of complex body parts including, but not limited to, limbs, tail, spinal cord, and portions of the heart [1,2,3,4,5,6,7,8,9,10,11,12,13,14,15,16,17,18,19,20,21,22]. These remarkable regenerative capacities have been studied for many years and more recent technological advances have begun to enable the identification of underlying molecular effectors and pathways. However, the regulatory circuitry that orchestrates these responses remains largely unknown.

Transcriptional profiling has revealed that many genes involved in basic limb and spinal cord development are actually reused, if and when those structures are regenerated [23,24]. These studies also confirmed that many of these developmental genes used by salamanders during regeneration show clear evolutionary conservation with those active during mammalian development. While these findings have supported the hypothesis that regeneration largely recapitulates specific portions of corresponding developmental processes, the key question of exactly how these events are reconfigured to be precisely adapted to the regenerative context remains open. Furthermore, the fact that some of the effector genes involved are conserved in mammals without conferring fully functional regenerative abilities indicates that important differences remain elsewhere, thus begging a closer examination of upstream regulatory molecules and mechanisms.

In this context, microRNAs represent strong candidates as important, possibly essential, regulators of regeneration pathways. Many microRNAs are highly conserved across metazoans and have been shown to be critically important in regulating both differentiation processes during development, and the subsequent maintenance of differentiated states in many cell types, including muscle, neurons, and skin [25,26,27,28,29,30,31,32,33,34]. Not surprisingly, then, differential expression of microRNAs has also been shown to be involved in many different types of cancer, emerging as valuable diagnostic tools for several different diseases [35,36,37,38,39,40].

More recently, microRNAs have been identified as playing important roles in regeneration across multiple different model systems. In zebrafish, miR-133 has been implicated in balancing the proliferation of blastema cells by regulating its target Mps1 kinase, thereby exerting a key role during fin and heart regeneration [41,42]. The transcription factor lef1, known for its role in Wnt signaling, which, itself, is centrally involved in zebrafish fin regeneration, has been shown to be regulated by miR-203 [43]. Additional examples of microRNA regulatory roles have also emerged in spinal cord and retinal regeneration in zebrafish [44,45,46,47,48].

In salamanders (newts and axolotls, the most studied in the regeneration field), microRNAs have also been shown to be involved in several different forms of regeneration. The let-7 family is critical for newt lens regeneration [37,39,49]. miR-128 was identified to play multiple roles in newt heart regeneration, including regulating non-myocyte hyperplasia and deposition of extracellular matrix [50] While miR-21 is involved in axolotl limb regeneration [51], let-7 has also been identified to be involved in axolotl tail and spinal cord injury regeneration and miR-196 is important in patterning the tail during regeneration [7,52,53].

Despite the fact that several interesting studies have emerged in recent years outlining the role of microRNAs in various regeneration paradigms in axolotl, we are still missing a more comprehensive survey of axolotl microRNAs. Particularly, an assessment of their degree of conservation with other vertebrates. To address this gap in knowledge we performed deep sequencing of small RNAs (less than 100 bps) in regenerating tail blastema *versus* control mature tail tissue. This approach allowed us to identify conserved microRNA families and also to identify putative novel microRNAs that we found especially enriched in the blastema sample. This study expands our knowledge of potential key regulators of gene expression during regeneration of highly conserved microRNAs but also identifies novel regulators that may be key evolutionarily distinct controllers of cells’ response to injury.

## 2. Results

### 2.1. Identification of Conserved MicroRNAs in Regenerating Tail Tissue

**Figure 1:**
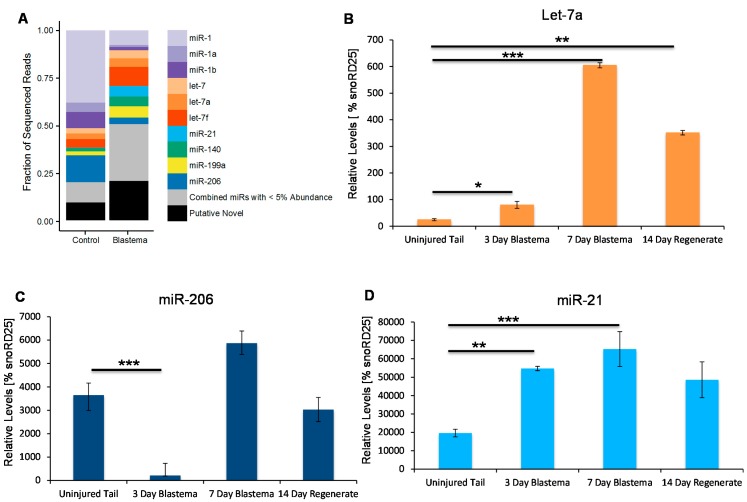
Deep sequencing of axolotl microRNAs. (**A**) Distribution of known and putative novel microRNA sequences in control tissue verses the three-day blastema. The fraction of reads that map to a known microRNA is shown for those microRNAs with at least 5% of the total number of sequenced reads. MicroRNAs present at less than 5% the total number of reads were collapsed into one category (gray portion). Putative novel microRNAs that did not map to mirBase 21 are also included in the total number of reads (black portion); (**B**) Quantitative RT-PCR analysis confirmed the deep sequencing data that let-7a is upregulated in the three day blastema and begin to return to basal levels by 14 days post injury (**C**) Quantitative RT-PCR analysis confirmed the deep sequencing data that miR-206 is downregulated in the three day blastema and returns to homeostatic levels by seven days post injury; and (**D**) Quantitative RT-PCR analysis confirmed the deep sequencing data that miR-21 is upregulated in the three day blastema, it stays elevated until seven days post injury and returns to basal levels by 14 days post injury. * *p* < 0.05, ** *p* < 0.01, *** *p* < 0.001.

In order to identify differentially-expressed microRNAs during regeneration we constructed two microRNA libraries: one from mixed tail tissues that have never regenerated (uninjured tail) and the second from an amputated tail three days post injury, a time point at which a distinguishable blastema has formed (Figure S1). Reads, 20–22 nucleotides in length, from each sample were mapped to known microRNA sequences present in mirBase 21, allowing up to two mismatches. This strategy identified 4564 conserved microRNA families (Figure 1A, Table S1).

We then validated expression of these known microRNA families using quantitative RT-PCR and in addition examined the dynamics of these microRNAs through the course of regeneration. Let-7a is a microRNA well-characterized to be involved in maintenance of the undifferentiated state in other model systems [38,39,54,55,56]. In axolotl tissue we see that let-7a is significantly upregulated in the early blastema tissue (Figure 1A). Its expression levels remain high during the early time points when it is known that a lot of cell proliferation occurs in the tail blastema and then its levels, although still elevated, begin to return to homeostatic levels by 14 days post injury when the cells in the blastema differentiate to replace lost structures (Figure 1B) [4,57].

miR-206 is an example of a microRNA that is found by deep sequencing to be downregulated in the early blastema (Figure 1A). We also validated this by qRT-PCR and show that it is downregulated three days post injury, but the levels increase again by seven days post injury. miR-206 has been characterized in other models to be highly expressed in muscle [35,36,58,59,60,61,62,63]. This early downregulation we see in axolotl may suggest that it plays a role in differentiation of cells in response to injury and its increase in expression seen by seven days post injury may indicate a time point at which cells in the blastema become specified again (Figure 1C).

miR-21 is an example of a microRNA that we found in very low abundance in the mature tissue library by deep sequencing but is highly enriched in the blastema sample (Figure 1A). By three days post-injury, miR-21 is highly upregulated and its levels stay high throughout the course of regeneration, just begin to return to homeostatic levels around day 14 (Figure 1D). miR-21 has previously been reported to be highly upregulated in the axolotl limb blastema where it regulated the levels of Jagged-1 [51].

### 2.2. Identification of Putative Novel MicroRNAs Enriched in the Tail Blastema

Analysis of the deep sequencing data also revealed a number of small non-coding RNA sequences that did not have homologies to other known microRNA.These putative novel microRNAs constitute a higher fraction of the total sequenced reads in the blastema sample than in the control sample (Figure 1A). To identify novel microRNAs expressed at different levels in the two samples, we clustered highly similar sequences into families and enumerated reads from each family present in the control and blastema samples (Table S2). To confirm the differential regulation of a subset of these putative novel microRNA families (Table S2) qRT-PCR primers were designed and the expression pattern was quantified over the course of tail regeneration.

**Figure 2:**
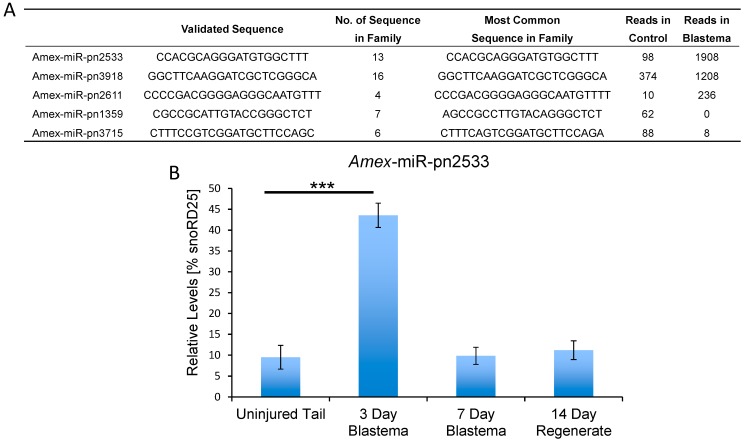
Identification of putative novel microRNAs in axolotl. (**A**) Table of putative novel microRNAs found in axolotl but not in other invertebrate or vertebrate species. The first three microRNAs are enriched in the blastema. The last two microRNAs listed are found in mature tissue but not at significant levels in the blastema. The table shows validated sequences, the number of sequences in the family and the number of reads found in the mature tissue *versus* the three-day blastema; and (**B**) Quantitative RT-PCR analysis confirmed the deep sequencing data that *Amex*-miR-pn2533 is upregulated in the 3 day blastema and returns to basal levels by seven days post injury (*** *p* < 0.001).

**Figure 3:**
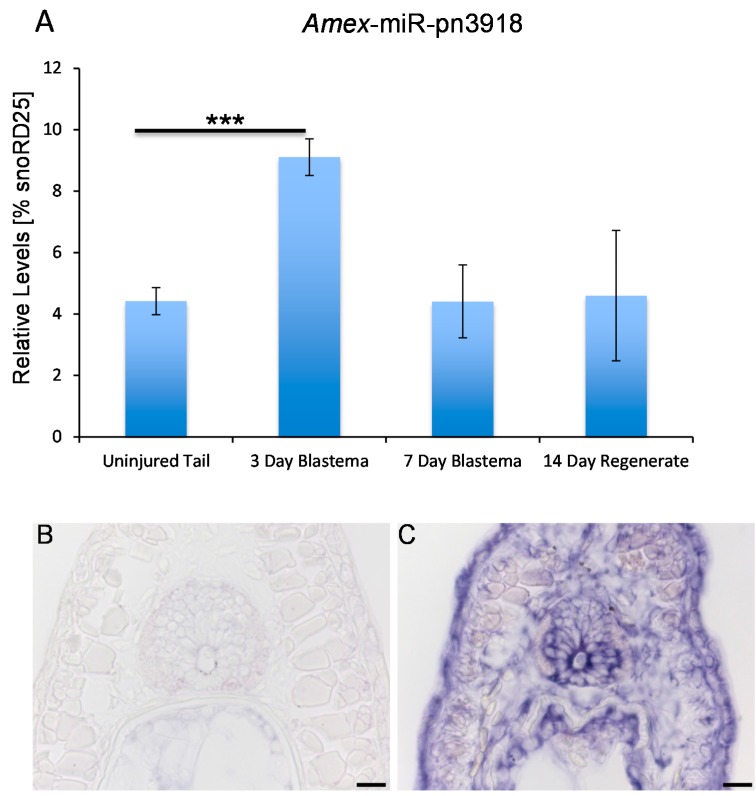
*Amex*-miR-pn3918 is upregulated in the tail blastema. (**A**) Quantitative RT-PCR analysis confirmed the deep sequencing data that *Amex*-miR-pn3918 is upregulated in the three day blastema and returns to basal levels by seven days post injury (*** *p* < 0.001); (**B**,**C**) *In situ* hybridization shows that *Amex*-miR-pn3918 is upregulated in cells distal to the plane of amputation, (**B**) is a section proximal to the plane of amputation, no positive staining is observed; and (**C**) Is a section distal to the plane of amputation; skin, radial glial, and blastema cells show positive purple staining for *Amex*-miR-pn3918 probe, *n* = 10, Scale bar = 50 µm.

We used qRT-PCR analysis to verify the deep sequencing data (Figure 2B and Figure 3A and data not shown). *Amex*-miR-pn2533 was in accordance with the deep sequencing data found to be enriched in the early blastema; however, its levels quickly return to basal levels by seven days post injury. This suggests that this microRNA may play a role in suppressing genes involved in maintenance of the differentiated state. A second putative novel microRNA, *Amex*-miR-pn3918 that we investigated more deeply is also found enriched in the blastema (Figure 2A) and also returned to homeostatic levels by seven days post injury (Figure 3A). Using *in situ* hybridization we found that this microRNA is not expressed in mature uninjured tissue (Figure 3B) but is expressed in many cell types during the early phases of regeneration, including skin cells, blastema cells, and neural stem cells (Figure 3C).

**Figure 4:**
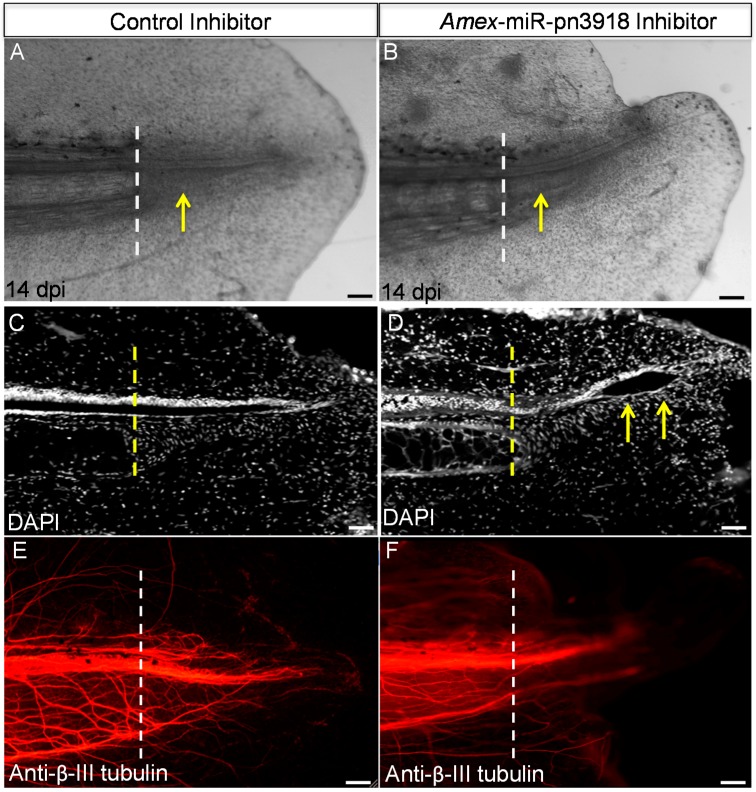
Inhibition of *Amex*-miR-pn3918 causes defects in tail regeneration injection of a control *versus* synthetic inhibitor of *Amex*-miR-pn3918 led to defects in skin regeneration (**A**,**B**). In addition defects in regeneration of the neural tube were visible, a terminal vesicle (yellow arrows) like structure was still present in the *Amex*-miR-pn3918 inhibitor animals 14 days post injury, but was no longer present in the controls (**C**,**D**). In addition, whole mount β-III tubulin staining revealed less axons to have regrown in the inhibitor treated animals (**E**,**F**). The dashed line in all panels indicates the original plane of amputation. Arrows in (**A**,**B**) indicate the regenerate, while in (**D**) arrows indicate the terminal vesicle structure still present. Control *n* = 10, Inhibitor *n* = 15. Scale bar = 100 µm.

**Figure 5:**
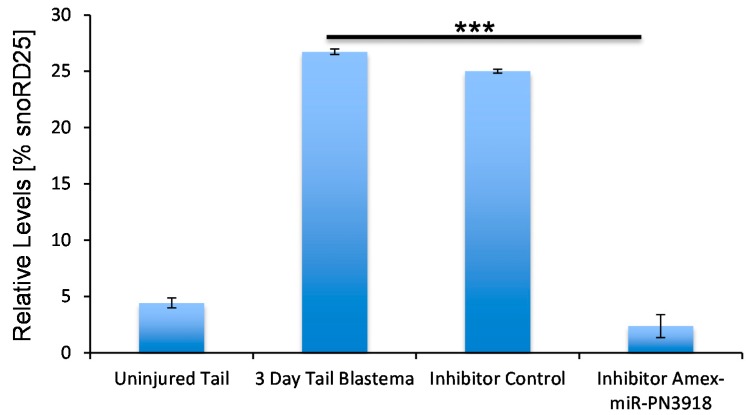
Quantitative RT-PCR analyses confirmed that the Amex-miR-pn3918 inhibitor significantly reduced the levels of Amex-miR-pn3918 in the three-day blastema. (*** *p* < 0.001).

To investigate the role this microRNA may be playing during regeneration we designed and microinjected a chemically-synthesized inhibitor of this microRNA that prevents the microRNA from binding to its target genes, into the early blastema. Inhibition of this microRNA resulted in an abnormal tail to be regrown with defects in the tail structures (Figure 4A,B). Whole-mount staining revealed that in the inhibitor-injected animals, a terminal vesicle-like structure remained at the end of the spinal cord (Figure 4C,D). The terminal vesicle is a transient structure that forms at the end of the spinal cord 3–4 days post injury; this zone is remodeled in the control animals by 7–10 days post injury (Figure 4C,D). In addition we found a defect in axonal regeneration in the inhibitor *versus* control animals. Whole-mount β-III tubulin staining revealed that fewer axons regenerated into the tail region in the inhibitor injected animals (Figure 4E,F). In the future it will be interesting to determine if this defect in axon regeneration is due to a lack of axon guidance molecule expression or due to a lack of proliferation of neural stem cells that differentiate into neurons to replace the lost neurons. To confirm that the inhibitor did in fact change the levels of *Amex*-miR-pn3918 qRT-PCR was performed on inhibitor-treated animals and showed that the levels were reduced (Figure 5); however, there is still some lower level expression of the microRNA, probably due to the fact that not all cells received the inhibitor. To ensure that the inhibitor was specific to *Amex*-miR-pn3918 we also quantified the levels of other microRNAs that differentially change in the three day blastema and found that their dynamics remained the same and were unaffected by inhibiting *Amex*-miR-pn3918 (Figure S2).

## 3. Discussion

This study is the first transcriptome analysis of microRNAs during axolotl tail regeneration. We identified 4564 conserved microRNA families from mature uninjured and three day regenerating tail tissue. Using qRT-PCR we confirmed the expression patterns for some of these conserved microRNAs initially identified from the deep sequencing data. We found that let-7a, a microRNA that has been implicated in maintaining cells in an undifferentiated state in many other model systems, especially in stem cells is found to be highly enriched in the three day blastema, peaking at its highest level in the seven-day blastema and returning to homeostatic levels by 14 days post injury (Figure 1A,B) [37,38,39,47,49,50,54,55,56]. This increased expression level in blastema cells corresponds to the time at which blastema cells are proliferating. It will be interesting to determine in the future if it is playing a role in maintaining blastema cells in an undifferentiated state.

Another conserved microRNA that we analyzed in more detail is miR-206, whose levels decrease in the early blastema in comparison to the uninjured tissue. This is a result we would expect as miR-206, like miR-1 whose levels also decrease in the early blastema, is a member of a microRNA family that is involved in maintaining muscle fibers in a differentiated state [35,36,58,59,60,61,62,63]. The blastema is made up of a mound of proliferating undifferentiated cells and, therefore, no markers of differentiated muscle have been found in the blastema. Therefore, we would expect to see very low levels of miR-206 or miR-1 in the blastema, as we have seen from our deep sequencing results (Figure 1A,C).

In addition, to identify a large number of conserved microRNAs we also identified a significant number of microRNAs that were not found by bioinformatics analysis to be present in any other genomes: these we have termed putative novel microRNAs (mir-pnXXX). As technology advances, and more sequence data from other model systems becomes available, we expect that these “putative novel microRNA” sequences may be found in other species. Many of these sequences were found to be enriched in the blastema tissue (Figure 2, Table S2), suggesting a potential important role for these in regeneration. We again used qRT-PCR to confirm the deep sequencing data. *Amex*-miR-pn2533 was found to be enriched by two days post injury in the blastema tissue in comparison to the control uninjured tissue and returned to normal levels by seven days post injury, again suggesting a possible role in suppressing genes involved in differentiation (Figure 2A,B). Another microRNA we found enriched in the blastema was *Amex*-miR-pn3918 (Figure 2A and Figure 3A,B). This microRNA shows similar dynamics to *Amex*-miR-pn2533, it is enriched in the early blastema and then returns to homeostatic levels at a time-point when cells in the blastema begin to differentiate. Using *in situ* hybridization, we found that several different cell types express this miR, including skin cells, radial glial cells, and cells in the blastema (Figure 3C). To further investigate the role of this microRNA during regeneration we injected a chemical inhibitor of the microRNA of interest into the early blastema. Control animals were injected with a control non-targeting inhibitor. Animals were examined 14 days post injury. In control animals a clear rod of cartilage was visible (Figure 4A,C), the neural tube had regrown (Figure 4C) and the axons had regrown into the regenerating tissue (Figure 4E). In comparison in the *Amex*-miR-pn3918 inhibitor injected animals defects were observed in the regrowth of the fin and of the neural tube (Figure 4C,D), a terminal vesicle-like structure was still visible in the Amex-miR-pn3918 animals. The terminal vesicle is a transient structure that is visible in the early stages of neural tube regeneration [7,53,57,64]. In addition, far fewer axons had regrown into the regenerating tail tissue and the shape of the fin structure had not returned to normal (Figure 4B,F). These multiple defects suggest that *Amex*-miR-pn3918 may target different genes in the different cell types in which it is expressed. It will be interesting in the future to elucidate the exact target of this microRNA in different cell types during regeneration.

In summary we have identified families of conserved and putative novel microRNA families in the axolotl during tail regeneration. We have verified our deep sequencing data using qRT-PCR for both conserved and putative novel microRNAs. In addition we have shown that one of these putative novel microRNAs has a functional role during regeneration, and perturbation of its normal expression levels leads to defects in the regenerate. Our findings open up new avenues in the study of the role of microRNAs in regeneration and will potentially uncover new regulatory circuitry essential for faithful regeneration in the future.

## 4. Materials and Methods

### 4.1. Animal Handling

All axolotls used in these experiments were bred at the Max Planck Institute of Molecular Cell Biology and Genetics (Dresden, Germany) or the University of Minnesota in accordance with the Institutional Animal Care and Use Committee (IACUC) No. 1411-32049A. Prior to all *in vivo* experiments animals (3–5 cm) were anesthetized in 0.01% *p*-amino benzocaine (Sigma, St. Louis, MO, USA). Tail amputations were performed using a sterile scalpel. Animals were placed in individual containers after surgery. Three days post tail amputation, a similar procedure was used to harvest the regenerating tissue, which was then frozen in liquid nitrogen.

### 4.2. Deep Sequencing of Axolotl MicroRNAs

Control and blastema samples were generated by pooling tail tissue from 20 mature axolotls that had either never regenerated or three days post injury. Total RNA was extracted with Trizol (Life Technologies, Carlsbad, CA, USA) according to the manufacturer’s protocol. Isolation of the small RNA fraction, generation of libraries, and subsequent deep sequencing was carried out by the Functional Genomics Library Construction Core at BC Cancer Agency Genome Sciences Centre, Vancouver, BC, Canada. The control and three day blastema samples were sequenced on the Illumina Genome Analyzer platform (Illumina, Inc., San Diego, CA, USA) to a depth of 17,878,944 and 21,685,056 reads, respectively, and analyzed with tools available in R/Bioconductor (PMID: 25633503). Reads were converted from Solexa format, adapter trimmed, and quality trimmed with ShortRead (1.26.0). Mapping to mirBase 21 was performed with Bowtie (1.1.2) allowing for up to two mismatches between the read and the template sequences. Rsamtools (1.18.2) was used to convert and sort reads into binary format and, subsequently, to enumerate reads for each known microRNA. Unmapped sequences occurring more than seven times in either sample were clustered by Levenshtein distance (number of insertions and mutations) and grouped into families based on a distance of four. To annotate each family, the most common sequence in each group was used to search for partial similarities to sequences in mirBase 21 with the Blast algorithm (NCBI Blast 2.2.31+). Sequences from this list were chosen for independent confirmation by qRT-PCR. Sequencing data have been deposited in the Gene Expression Omnibus repository under accession code GSE72057 and details of the bioinformatics analysis can be found at https://github.com/micahgearhart/amex-miR.

### 4.3. Quantitative Real-Time PCR

For each sample tissue samples were taken from 10 animals and pooled. RNA samples were extracted using standard Trizol techniques (Invitrogen, Grand Island, NY, USA). Genomic DNA was removed from RNA by treatment with DNase for 30 min at 37 °C. The DNase was inactivated by adding 25 mM EDTA and heating to 65 °C for 15 min. The miScript II RT kit from Qiagen (Hilden, Germany) was used for cDNA synthesis. The qRT-PCR was carried out using the Qiagen miSCript SYBR Green PCR kit. Qiagen designed primers compatible with the miScript kit were purchased to quantify conserved microRNAs and custom designed primers were made by Qiagen to amplify the corresponding putative novel axolotl microRNAs. All Qiagen kits were used according to the manufacturer’s protocols. Triplicates of qRT-PCR experiments were carried out.

### 4.4. In Situ Hybridization

Three days after injury, tissue samples were collected and fixed in freshly made 4% PFA with 0.01% glutaraldehyde (Sigma) in PBS for 3 h. After fixation, samples were washed three times in PBS for 5 min and in PBST three times for 5 min. Then, tissue samples were incubated for 10 min in 50:50 PBS:30% sucrose in PBS, and overnight in 30% sucrose in PBS, before embedding the samples in Tissue Tek (Sakura, Torrance, CA, USA). Frozen tissue samples were cut into 20-m thick longitudinal sections.

Tissue sections were hydrated in PBS for 5 min, treated with denaturation buffer (2% SDS and 100 mM DTT in PBS) for 20 min, and washed three times in PBST for 5 min. The slides were digested with 2 g/mL proteinase K in PBS for 5 min, and postfixed in 4% PFA for 10 min. Then, slides were washed three times with 0.1% Tween-20 in PBS for 5 min, treated with triethanolamine buffer for 10 min (100 mM triethanolamine and 0.25% acetic anhydride in water), and washed again three times with PBST for 5 min.

Before hybridization, slides were treated with hybridization solution (65% formamide, 5 SSC, 50 µg/mL heparin, 0.1% Tween-20, and 0.5 mg/mL tRNA) for 15 min at 70 °C. The probes were diluted in hybridization buffer (500 ng/mL) and denatured for 10 min at 75 °C. Dioxygenin-labeled probes against microRNAs of interest and control probes were purchased from Exiqon (Vedbaek, Denmark). Hybridization was carried out overnight at 50 °C. The next day, slides were incubated in five SSC pre-warmed to 70 °C for 5 min at room temperature, and in 0.2 SSC for 1 h at 70 °C. Then, slides were treated with Solution B1 (0.1 M Tris, pH 7.5, and 0.15 M NaCl in water) for 10 min, and then with blocking buffer [10% fetal calf serum (Sigma) and 0.05% Tween-20 in Solution B1] at room temperature for 1 h. Alkaline-phosphatase associated anti-digoxigenin antibody (Roche, Indianapolis, IN, USA) was diluted 1:2000 in blocking buffer, and slides were incubated in the antibody solution overnight at 4 °C. The next day, slides were washed three times with PBST for 10 min, once with PBST for 5 min, and three times with freshly prepared alkaline phosphatase (AP) buffer (100 mM Tris-HCl, pH 9.5, 50 mM MgCl_2_, 100 mM NaCl, and 0.1% Tween-20 in water) for 5 min. Finally, slides were incubated with AP substrate (0.5% NBT (Roche) and 0.375% BCIP (Roche) in AP buffer) in the dark until development of a blue/purple color. Slides were rinsed with PBST several times, and a drop of 0.5 M EDTA was added to the top of the slides to stop the reaction. Slides were mounted with 80% glycerol and imaged using a Olympus Microscope (Center Valley, PA, USA) at 10× or 20× magnification.

### 4.5. MicroRNA Inhibitor Injection

Inhibitor injections were carried out as previously described in Erickson and Echeverri 2015 [65]. Briefly microRNA inhibitors against putative novel microRNAs were custom synthesized by Qiagen. Control and *Amex*-miR-pn3918inhibitor were diluted to final concentration of 10 µm in PBS plus Fast Green. World Precision Instruments pressure injector was used to inject the solutions directly into the tissue directly before amputation and two days post amputation into the blastema.

### 4.6. Whole-Mount Staining

At 14 days after injury, tissue samples were collected by amputating the tail of anesthetized axolotls at the level of the hind limb. The tissue samples were fixed in freshly made 4% paraformaldehyde (PFA) (Sigma) in PBS for 1 h. To improve the antibody penetration of the tissue, samples were washed three times with 0.1% Tween-20 (Sigma) in PBS (PBST) for 5 min, digested with 20 mg/mL proteinase K in PBS for 30 min, and post fixed in 4% PFA for 10 min. The samples were washed with PBST three times for 5 min, and then washed three times with 0.2% Triton X-100 (Sigma) in PBS (PBSTX) for 10 min. To prevent non-specific binding of the antibodies, the samples were blocked in 10% goat serum (Sigma) in PBSTX for 1 h at room temperature. Mouse anti-b-III-tubulin primary antibody (Sigma) was diluted 1:1000 in blocking buffer, and samples were incubated in the antibody solution overnight at 4 °C. The next day, unbound antibody was eliminated by washing the tissue samples four times in PBST for 30 min. The secondary antibody (goat anti-mouse Alexa Fluor 568, Invitrogen) and DAPI were both diluted to a final concentration of 1:200 in blocking buffer, and the samples were incubated in the secondary antibody for 1 h at room temperature. To eliminate the unbound antibody, samples were washed four times in PBST for 30 min. Prior to imaging, samples were cleared with 1:2 benzyl alcohol:benzyl benzoate (BABB) (Sigma). Tissue samples were dehydrated by incubating in 50:50 PBS:methanol for 10 min and 100% methanol twice for 10 min. The samples were then incubated in BABB for 10 min, and mounted onto a cover slip using BABB as the mounting medium. Images were captured using an inverted Leica DMI 6000B Microscope (Wetzlar, Germany) at 10× magnification. All incubations and washes were carried out at room temperature unless indicated otherwise.

## Supplementary Materials

Supplementary materials can be found at http://www.mdpi.com/1422-0067/16/09/22046/s1.
