# Supplementary material for: Identification of Conserved and Novel MicroRNAs during Tail Regeneration in the Mexican Axolotl
Source: Int J Mol Sci. 2015 Sep 11;16(9):22046–61. doi: 10.3390/ijms160922046 (PMC4613296; doi:10.3390/ijms160922046)
Supplement: Supplementary file 1 [file ijms-16-22046-s001.zip › ijms-94898-Supplementary Information/ijms-94898-Supplementary Figures.pdf]

Supplementary Information

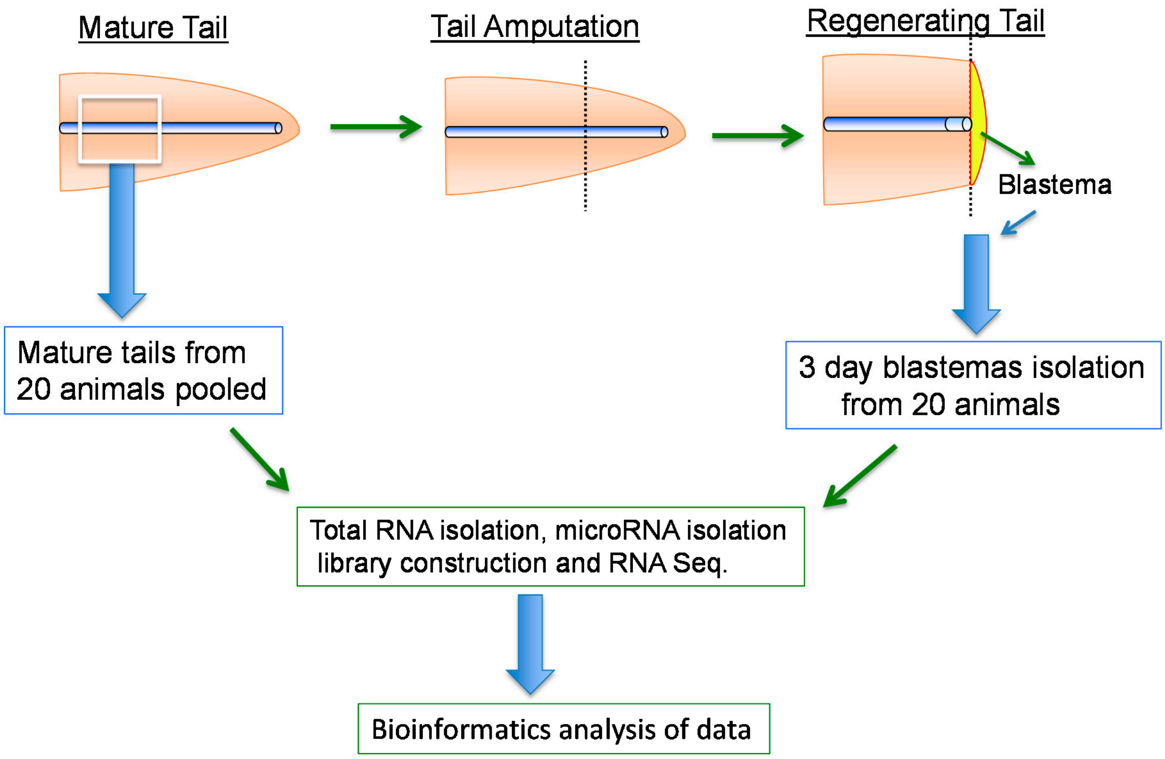

Figrue S1. Schematic diagram outline where the tissue samples were collected from to construct the libraries used for deep sequenching of axolotl microRNAs. Tissue samples were collected from a tail region of 20 axolotls to construct the mature tissue library. Three day blastema samples were collected from 20 axolotls and RNA was extracted from the pooled tissue samples.

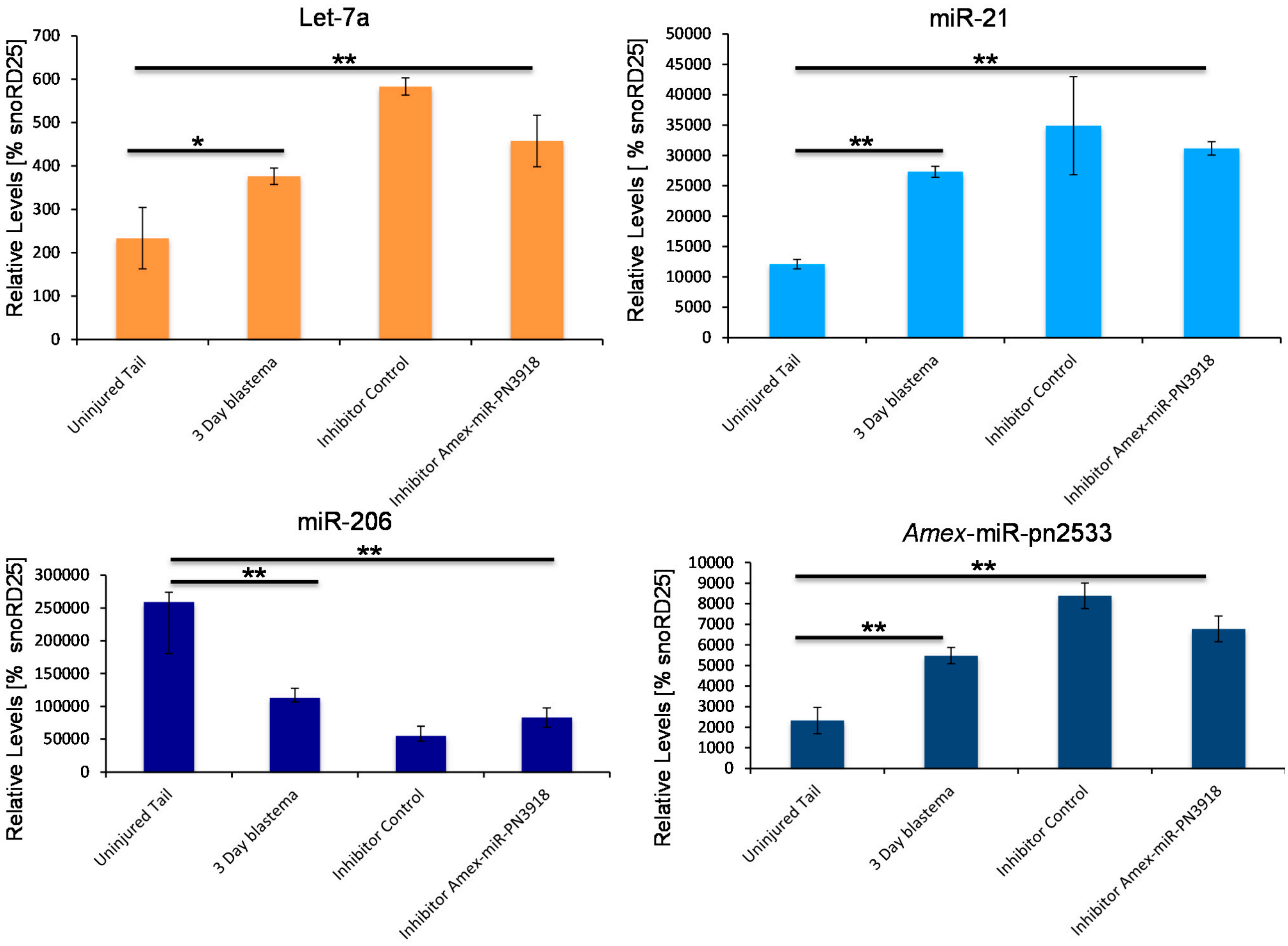

Figrue S2. Quantitative RT-PCR analyses confirmed that the Amex-miR-pn3918 inhibitor did not affect the regulation of other microRNAs know to be differentially regulated in the 3 day tail blastema. Levels of let-7a, miR-21, miR-206 and Amex-pn2533 were quantified in uninjured tails, 3 day blastemas *versus* inhibitor control and Amex-miR-pn3918 inhibitor injected animals 3 days post injury. (* $p < 0.05$, ** $p < 0.01$). $n = 3$.
